# The Effects of *Medicago Sativa* and *Allium Porrum* on Iron Overload in Rats

**DOI:** 10.5539/gjhs.v7n7p137

**Published:** 2015-04-23

**Authors:** Ali Mirzaei, Hamdollah Delaviz, Mahsa Mirzaei, Mohsen Tolooei

**Affiliations:** 1 Medicinal Plants Research Center, Yasuj University of Medical Sciences, Yasuj, Iran; 2 Cellular and Molecular Research Center, Faculty of Medicine, Yasuj University of Medical Sciences, Yasuj, Iran; 3 Department of Biology, Islamic Azad University, Ahar branch, Ahar, Iran

**Keywords:** *Medicago sativa*, *Allium porrum*, iron overload, iron dextran, Yasuj

## Abstract

**Purpose:**

Iron overload may occur due to regular blood transfusions and high intestinal iron absorption. Currently, there is no effective drug without side effects for the treatment of iron excess in thalassemia and other iron storage diseases, except chelation therapy, which is the only safe method for iron excretion. Thus, scientists are more focused on medicinal plants rich in phytochemical compounds for the removal of iron in thalassemia. Therefore this study was managed to discover the therapeutic potential of hydro- alcoholic extract of *Allium porrum* and *Medicago sativa* for iron chelating potential.

**Methods:**

Aerial parts of *Allium porrum* and *Medicago sativa* werecollected in Yasuj Iran. Rats were divided into seven groups each containing six. Extracts were administrated in four groups (two groups for each extract) by single doses of each plant with 200 and 400 mg / kg body weight by (i.p.) route every other day for28 days. Group 1 as negative control received saline (0.5 ml/kg) by (i.p.) route. Positive control received iron dextran 200 mg/kg body weight. Experimental groups 1 and 2 for each plant extract were fed with 200 and 400 mg/kg, hydro-alcoholic extract respectively via (i.p.) route, 1 h after the injection of iron dextran. Standard group was treated with deferoxamine (DF) 50 mg/kg by (i.p.) route1 h after the injection of iron dextran. Serum iron (SI) and serum total iron binding capacity (TIBC) were determined. The serum ferritin was then measured using enzyme immunoassay ELISA kit for rat. For Analysis of data ANOVA test was used.

**Results:**

Hydro-alcoholic extract of *Medicago sativa* and *Allium porrum* at 400 mg/kg showed significant (p<0.05) iron chelating activity compared to control. The plant extracts with dose 200 mg/kg also reduced the iron and ferritin content but the effect was lower level compared to higher doses. The plant extract effects were similar to that of standard drug deferoxamine. Iron and ferritin levels were significantly reduced in experimental groups when compared to positive group especially in Medicago sativap<0.05. There was no difference between standard drugs and last concentration of plant extracts.

**Conclusion:**

protective effect of *M. sativa* and *A. Porrum* against iron overload in rat model was reported. Significant decrease in serum ferritin and iron concentration was reported in iron overload rats which induced by iron dextran.

## 1. Introduction

β- Thalassemia major is a congenital disease that is characterized by severe anemia. It depends on regular blood transfusion in early life stage. The disorder is more prevalent in Asia particularly in Mediterranean countries (Flint, Harding, Boyce, & Clegg, 1998). In major β-Thalassemia hemoglobin precipitation occurs and consequently induces reactive iron and free radicals in blood circulation. Iron overload may occur due to regular blood transfusions and high intestinal iron absorption (Milena, 2010).

In human body iron excretion mostly has one directional cycle, thereby no repelling by excretory system. Consequently, iron excess gets deposited and disturbs many physiological functions in heart, liver, brain, spleen and endocrine systems (Taher, Isma’eel, & Cappellini, 2006; Rund & Rachmilewitz, 2005; Loukopoulos, 2005).

Nowadays, synthetic iron chelators are used for iron excretion in iron overload state. Chelators are small molecules that form a powerful complex with metal ions in organisms so they are called iron binders in literatures. Iron chelation is recommended after 10-20 blood transfusion practices or when amount of serum ferritin gets more than 1000 mg/ml (Thalassemia International Federation, 2008).

Iron chelating method is a useful assay for estimation of plant chelating potential. Chelating activity of medicinal plants mostly depend on its phytochemical constituents especially phenolic substances (Gupta et al., 2011).

Deferoxamine is a synthetic iron chelating drug used to prevent the increase of iron concentration in plasma. Excessive use of deferoxamine by injection route has limited the drug application in clinics. Moreover, its short life span has restricted its efficiency in practice (Borgna-Pignatti & Galanello, 2004; Yasser et al., 2008).

Deferiprone is another iron chelator which is used in iron overload treatment. It is also applicable for treatment of myocardial siderosis in iron toxicity. Its long timed application causes a reduction in white blood cell count mainly neutrophils.

Currently, there is no effective drug without side effects for the treatment of iron excess in thalassemia and other iron storage diseases, except chelation therapy, which is the only method for iron excretion. Thus, scientists are more focused on medicinal plants rich in phytochemical compounds for the removal of ironin thalassemia.

*Medicago sativais* (family:Fabaceae) which its sprouts are often consumed as salad vegetable. Plant contains total phenol, flavonoids, alkaloids, coumarins, triterpenes and phytosterols. *Medicago sativa* possesses different potential such as antioxidants, antidiabetics, anti-rheumatic, Anti-cancer, cardio tonic activity and lowering cholesterol capacity (Rana et al., 2010; Kundun, 2012).

*Allium porrum* (family: Alliaceae) is rich source of vitamins C, E, B, potassium, iron and copper. It also contains carotenoids chlorophyll, glycosides, andtotal phenols and flavonoid. It has been used in treatment of blood clotting diseases (Rana & Noora, 2012, Cropandfood, 2007). In vitro iron chelating potential of these plants were detected in our laboratory; therefore this study was managed to discover the therapeutic potential of hydro- alcoholic extract of *Allium porrum* and *Medicago sativais* in iron chelating potential.

## 2. Materials and Methods

### 2.1 Collection and Extraction of Plant Material

Aerial parts of *Allium porrum* and *Medicago sativa* were collected in Yasuj Iran during the month of June, 2012. Samples were identified, authenticated and a voucher specimen (No. MPRC-YUMS-13) has been deposited in the medicinal plants research center at Yasuj University of Medical sciences. Hydro- alcoholic extract was prepared by maceration process. Extractions were concentrated using rotary evaporator (Heideolph model 4000; Germany) to obtain a dry extract.

### 2.2 Determination of Total Phenolic Compounds

The total phenoliccontent of extract was determined (Karim et al., 2011).

### 2.3 Antioxidant Activity of Dipheny-picrylhydrazyl (DPPH)

The antioxidant activity of extract was assessed by (Mirzaei & Mirzaei, 2013) method.

### 2.4 Metal Chelating Activity

The chelation potential of Fe^2+^ ions was determined by extracts, using modified method of Dinis (Dinis et al., 1994).

### 2.5 Experimental Protocol

Rats were randomized in to seven groups each containing six. Rats were in a controlled environment with 22 ± 2 °C temperature, 65 to 70% humidity, and 12 h light/dark cycle. They were fed with standard laboratory diet (Pars, Iran Ltd., Tehran, Iran). Treatment followed in seven groups (two groups for each plant extract) by single doses, every other day for 28 days. Group 1 as a negative control received normal saline (0.5 ml/kg) i.p. and Positive control received iron dextran (Vifor Inc., Switzerland) 200 mg/kg b.w. (Nematbakhsh, 2013) by i.p. route.

Experimental groups 1 and 2 for each extract were fed with 200 and 400 mg/kg, hydro-alcoholic extract respectively according to LD_50_ of plant extracts via i.p. route, 1 h after the injection of iron dextran. Standard group was treated with deferoxamine (DF) 50 mg/kgi.p.1 h after the injection of iron dextran. At the end of the study, animals were exsanguinated under diethyl ether anesthesia. Blood samples were collected by heartpuncture and serum was separated for biochemistry tests. Serum samples were stored after centrifugation.

The levels of serum iron (SI) in and serum total iron binding capacity (TIBC) were determined using quantitative diagnostic kits (Pars Azmoon, Iran) in acid wash tubes. The serum ferritin was then measured using enzyme immunoassay ELISA kit for rat (Immunology Consultants Laboratory Inc., USA).

### 2.5 Statistical Analysis

All data were expressed as mean ± SD. For Analysis of data ANOVA test was used for the comparison. P<0.05 was described as significant whereas p<0.001 was identified as highly significant.

## 3. Results

**Figure 1:**
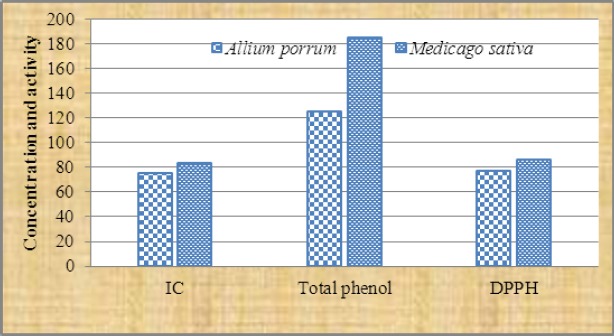
Total phenolic content ¸DPPH activity and Iron chelating potential in in *Medicago sativa* and *Alliumporrum* IC= Iron chelating DPPH = Dipheny-picrylhydrazyl.

According to Figure 1 *Medicago sativa* extract was reported with the maximum total phenolic content, antioxidant and iron chelating activities. The hydro-alcoholic extracts of *Medicago sativa* and *Allium porrum* were found to contain 185 ± 11.6 and125±10.8 mg Gallic acid equivalent/g of phenolic respectively though, the iron chelating activity were 83 ± 9.1 and 75.5 ± 5.9 % at 4mg/ml (Figure 1).

**Figure 2:**
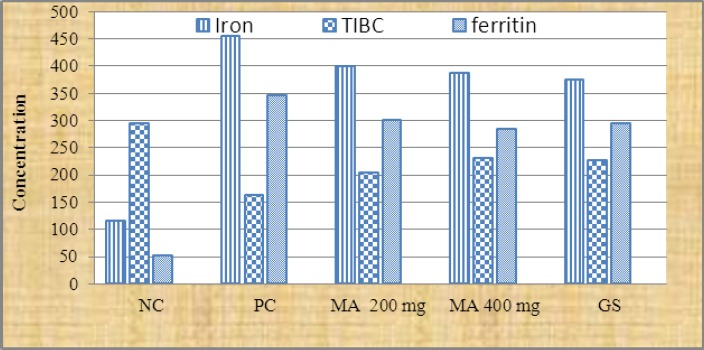
Iron, TIBC and ferritin content in iron overload rats which administrated Medicago *sativa* NC= negative group; PC = positive group; MA= *Medicago sativa*; GS = standard group; TIBC = Total Iron Binding Capacity; n= 6.

**Figure 3:**
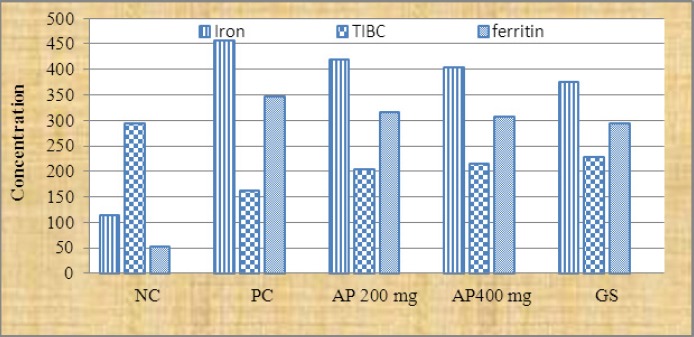
Iron, TIBC and ferritin content in iron overload rats which received Allium *porrum* in different groups NC= negative group; PC = positive group; AP= *Allium porrum*; GS = standard group; TIBC = Total Iron Binding Capacity; n= 6.

A significant diffrence was recorded between phenolic content in *Medicago sativa* and *Allium porrum* extracts P<.001. However, the other two tests antioxidant and iron chelating activities were similar in both plants (Figure 1). The administration of hydro-alcoholic extract of *Medicago sativa* and *Allium porrum* extract (200 mg/kg and 400 mg/kg) reduced the iron concentration to 399.7 ± 5.4, 386.3 ± 6.9 and 418.8 ± 8.6, 404.3 ±7.1 respectively after 28 days. However deferoxamine (DF) 50 mg/kg reduced the iron level to 375.3±6.6 (Figure 2, 3).

Hydro-alcoholic extract of *Medicago sativa* and *Allium porrum* at 400 mg/kg showed significant (p<0.05) iron chelating activity compared to control. The plant extracts with dose 200 mg/kg also reduced the iron and ferritin content but the effect was slow compared to higher doses. The plant extracts effect was similar to that of standard drug deferoxamine. Iron dextran induced iron overload, in all of the groups and showed a significant (p<0.001) increase in serum iron, serum ferritin and decreased in TIBC concentration as compared to control group after 28 days (p<0. 01) (Figure 2, 3).

**Figure 4:**
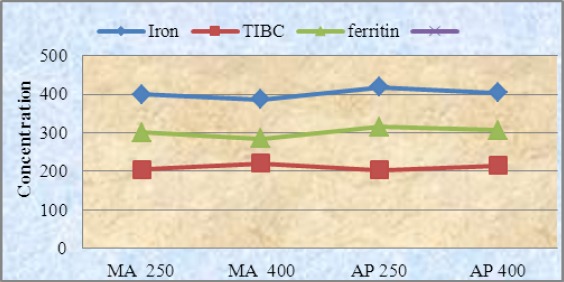
The comparison of Iron, TIBC and ferritin content in iron overload rats which received *Medicago sativa* and *Allium porrum* in different dossages NC= negative group; PC = positive group; AP= *Allium porrum*; MA= *Medicago sativa*; GS = standard group; TIBC = Total Iron Binding Capacity; n= 6.

Iron and ferritin levels were significantly reduced in experimental groups when compared to positive group especially in *Medicago sativa* p<0.05. No significant difference was reported between standard drug and last concentration of plant extracts. The analysis revealed no statistically significant difference between *Medicago sativa* and *Allium porrum* extracts in terms of iron chelating (Figure 4).

## 4. Discussion

Plants with medicinal capacity have always been an important target for drug development. It is important that the *M. sativa* and *A. porrum* extracts produced a significant dose dependent on chelating potential incomparison to control. The present result indicated that *M. sativa* and *A. porrum* at the dose of 200 and 400 mg/kg reduced the iron and ferritin concentrations. Iron and ferritin levels were significantly increased after injection of iron dextran in 28 days. This expected results supported the iron overloading process in present study.

Medicinal plants are rich sources of natural phytochemicals with antioxidant activity that are mainly associated with phenolic compounds (Piett, 2000). Generally, plant extracts with high levels of phenolic compounds revealed a good iron chelating activity; therefore such kind of herbal extracts can be used as a choice of chelator for treatment of thalassemia in future (Ebrahimzadeh et al., 2008d). In present study there was a direct correlation with iron chelating and antioxidant activity and phenol contents.

Iron at normal range is necessary for the metabolic pathways in body. However, in major β-thalassemia which is the best case of iron overload, reactive oxygen species are mostly formed by deposition of iron excess in vital organs. They usually used DF as an iron chelator for removal of iron excess (Milena, 2010). At the present study *Medicago sativa* and *Allium porrum* as iron chelators significantly decreased iron in animal groups by forming soluble and stable forms by interactions with flavonoids after incubation (Mira et al., 2002).

Iron concentration was significantly reduced in experimental groups compared to control serums P<0.001. Chelating potential of the extractsmay be related to a number of possible mechanisms. Experimental studies have shown thatphytochemical content and antioxidant activity isassociated withchelating property (Mira et al., 2002).

It also has been reported that flavonoids in plant extracts can interact with iron in live organisms (Mira et al., 2002). Therefore, it may be suggested that chelating property of the hydro – alcoholic extract of *M. sativa* and *A. porrum* might be due to the presence of flavonoid substances. In the recent work a significant reduction was reported in serum iron and ferritin rate, by *Medicago sativa* and *Allium porrum* extracts respectively P<0.001.*Medicago sativa* extract showed more potential but had no significant effect on iron and ferritin concentration in experimental group compared to *Allium porrum* extract p<0.05. This difference could be due to antioxidant potential and phytochemical contents.

Iron overload is the main cause of mortality in major β-thalassemia because it disturbs function of vital organs such as heart and brain. Thus, removal of iron is essential for their quality of life. In present paper *M. sativa* and *A. Porrum* extracts were optimal chelator for iron reductin at in vivo state. *M. sativa* and *A. Porrum* were effective plant chelators to reduce the iron in an iron overload model.

Present result confirmed that *M. sativa* with the dose of 400 mg/kg was potentially more protective than *A. porrum* against iron overload. This results need to be confirmed in serial experimental researches with different concentration of iron overload. The data obtained in recent study indicated that *M. sativa* and *A. porrum* demonstrate an iron chelation effect in reducing the serum iron level. Iron chelators can bind with ion metals such as calcium and magnisum which are vital ions for live organisms. The prolong use of plant chelators with high concentration may decrease calcium and magnisum levels. A new project or identify of calcium and magnisum concentration is essential.

## 5. Conclusion

In this study protective effect of *M. Sativa* and *A. Porrum* against iron overload in rat model was reported. Significant decrease in serum ferritin and iron concentration were reported in iron overload rats which induced by iron dextran.
